# Deep learning–based histologic classifiers enable molecular subtyping of metastatic prostate cancer

**DOI:** 10.1172/jci.insight.201872

**Published:** 2026-08-06

**Authors:** Zhijun Chen, Erolcan Sayar, Daniela Guevara, Helen Richards, Haoyue Zhang, Radhika A. Patel, Agnes C. Gawne, Lucas J. Liu, Ilsa Coleman, Ruth Dumpit, Colm Morrissey, Michael T. Schweizer, Ruben Raychaudhuri, Laura S. Graham, Evan Y. Yu, Heather H. Cheng, Chien-Kuang C. Ding, Yuzhuo Wang, Peter Choyke, Baris Turkbey, Chantal Chanel-Vos, Christina Fedorov, John R. Otilano, Troy Kane, Jyothi Manohar, Michael Sigouros, Jones T. Nauseef, Ana Molina, David Nanus, Scott T. Tagawa, Juan Miguel Mosquera, Himisha P. Beltran, Ruth Etzioni, Peter S. Nelson, Rama Soundararajan, Ana M. Aparicio, Cora N. Sternberg, Michael C. Haffner, Stephanie A. Harmon

**Affiliations:** 1 National Cancer Institute, NIH, Bethesda, Maryland, USA.; 2 Fred Hutchinson Cancer Center, Seattle, Washington, USA.; 3 Englander Institute for Precision Medicine and Weill Cornell Medicine, New York, New York, USA.; 4 University of Washington, Seattle, Washington, USA.; 5 UCSF, San Francisco, California, USA.; 6 University of British Columbia, Vancouver, British Columbia, Canada.; 7 The University of Texas MD Anderson Cancer Center, Houston, Texas, USA.

**Keywords:** Clinical Research, Oncology, Biomarkers, Machine learning, Prostate cancer

## Abstract

Metastatic prostate cancer is a clinically and molecularly heterogeneous disease. Under the selective pressure of androgen receptor–directed (AR-directed) therapies, resistant phenotypes frequently emerge, posing significant diagnostic and therapeutic challenges. Neuroendocrine prostate cancer (NEPC) is a clinically important phenotype characterized by lineage plasticity, neuroendocrine features, visceral metastases, and poor prognosis. Accurate diagnosis of NEPC remains difficult owing to its histologic and molecular complexity but has high clinical relevance. In this study, we developed a deep learning model that leverages interpretable cellular features to improve feature extraction from H&E-stained tissue sections (NEURAL-PC). By incorporating a multiple-instance learning framework, NEURAL-PC enables robust NEPC classification solely from H&E tumor images, achieving an area under the receiver operating characteristic curve of 0.921 in independent external validation. In addition to its diagnostic utility, NEURAL-PC provides prognostic information that enables further subclassification of advanced prostate cancer across diverse datasets, supporting its strong prognostic value and generalizability. Broadly, our work highlights a hybrid approach that integrates features across different domains, offering a promising strategy for developing reliable deep learning tools in pathology. Built on this framework, NEURAL-PC represents an extensively validated diagnostic and prognostic model for advanced prostate cancer.

## Abstract 2

Deep-learning based tools can provide quantitative assessment of molecular phenotypes in metastatic prostate cancer biopsies

## Introduction

Prostate cancer (PC) is the most common non-cutaneous malignancy in men in the United States (1). While localized PC is often curable, metastatic disease is associated with high mortality and remains challenging to treat (2). Importantly, metastatic PC is highly heterogeneous, and over the past decade, several clinically relevant phenotypes have been identified (3–13). However, accurately diagnosing these phenotypes remains difficult and represents a clinical challenge (4). Among the phenotypes encountered in patients with advanced disease, high-grade neuroendocrine prostate cancer (NEPC) is particularly notable. NEPC exhibits morphologic features overlapping with small-cell carcinomas, though its histologic appearance is variable and difficult to interpret (14, 15).

Broadly, NEPCs with or without small-cell morphology are increasingly observed in patients who progress on therapies targeting the androgen receptor (AR) signaling axis (4, 10, 16, 17). These treatment-emergent NEPCs are typically associated with aggressive clinical behavior, poor response to further AR-directed therapy, and a dismal prognosis (12, 15, 16, 18–20). Transcriptomic profiling distinguishes these tumors from conventional AR-driven prostate adenocarcinomas (12, 13, 21–24). They frequently express high levels of stem cell and progenitor as well as neural and neuroendocrine markers, reflecting their profound lineage plasticity.

Despite these defining molecular features, accurate diagnosis of NEPC remains challenging — even for expert pathologists — owing to the marked histomorphologic diversity of these tumors. Notably, a subset of PCs displays clinical and molecular similarities to NEPC but lacks overt histologic features of high-grade neuroendocrine carcinoma (4, 11, 14, 16, 18, 19, 25–27). Ancillary studies, such as immunohistochemical (IHC) staining for neuroendocrine markers, are not universally available and are often applied in a non-standardized manner (4, 15), resulting in inconsistent detection and reporting of NEPC. Moreover, neuroendocrine marker positivity alone does not always correlate with the aggressive clinical behavior characteristic of true NEPC (3, 19). This can lead to diagnostic misclassification and can complicate clinical interpretation.

These limitations underscore the urgent need for a robust, standardized, and reproducible diagnostic assay to accurately identify NEPC and enable consistent detection of this aggressive PC variant. From a clinical standpoint, such a test would be of substantial value, as it would streamline the diagnostic process, allowing for accurate and timely treatment decisions in patients who require more intensive therapeutic intervention involving platinum-based chemotherapy — often within a narrow window of treatment opportunity (16, 18, 25, 26).

With the recent development of imaging devices and machine learning algorithms, tasks like tumor detection, segmentation, and classification in digital pathology can be addressed by various deep learning models (28–30). However, the training of models for such applications usually requires a large number of manually annotated images. Collecting high-quality data and annotations is usually difficult since it requires well-trained pathologists and is time-consuming. Thus, the development of unsupervised or semi-supervised methods has become popular (31–33). A convolutional autoencoder (CAE) is a type of neural network composed of an encoder that uses convolutional layers to extract low-dimensional image features, and a decoder that employs transposed convolutional layers to reconstruct the original image from these features (34). Variants of CAEs have shown promising performance across a range of medical imaging tasks (33, 35–38). However, many CAE-based models face challenges related to feature interpretability and are susceptible to overfitting (39). Prior work has attempted to address these limitations by introducing sparsity constraints (40, 41) or modifying the objective function to regularize the latent space (42). While such strategies can enhance model performance, it remains difficult to directly relate latent features to human-interpretable characteristics.

In this study, we test the hypothesis that image-based machine learning methods can robustly detect NEPC. To this end, we developed a CAE-based model to detect NEPC by improved feature interpretability and robustness, termed NEURAL-PC (NEUroendocrine Recognition Using Attentive Learning in Prostate Cancer). NEURAL-PC integrates biologically meaningful cellular features into the learning process through a hybrid architecture. Specifically, we introduce a projection module that maps the encoder’s output to a predefined latent space informed by cellular features. This projection acts as a form of regularization and enhances the quality of image feature extraction. A downstream classifier built on top of our pre-trained model outperforms benchmark approaches in detecting NEPC. Validated within external cohorts, NEURAL-PC shows strong generalizability and relevance to clinical diagnostics and decision-making.

## Results

### Initial development of NEURAL-PC.

NEURAL-PC extracts features from standard H&E images using a CAE that is regularized, i.e., penalized during the learning process, by handcrafted cellular features (Figure 1; see Methods for additional details). The model was first pre-trained in an unsupervised manner from a diverse development cohort of metastatic PC specimens obtained from the University of Washington Rapid Autopsy (UW-RA) cohort (43, 44). This cohort comprises 722 tissue microarray (TMA) cores taken from 204 metastatic sites of 52 patients, representative of the molecular and morphologic heterogeneity (including sarcomatoid and squamous features) of advanced PC (4, 43, 44). All samples have undergone comprehensive histologic and molecular characterization including expert histopathology review for reference standards in model development (45–47) (see Methods for additional study cohort details and NEPC classification reference standard guidelines). We utilized 502 cores (450 for training and 52 for validation) from 31 patients for training, and reserved the remaining cores from 21 patients as a testing set.

Cores were pre-processed in tiles of size 250 × 250 pixels at ×40 magnification through an automated pipeline to identify cancer areas (Figure 1A and Methods). Cellpose (48) was used to segment nuclei and extract a cellular feature vector (49) for each tile (Figure 1B), which contained information such as nuclear size, shape, texture, and distribution. The objective of the pre-training step was to reconstruct images and corresponding handcrafted cellular features, as shown in Figure 1C, with further architecture details in Supplemental Figure 1 (supplemental material available online with this article; https://doi.org/10.1172/jci.insight.201872DS1). The model inherently optimizes image features to incorporate interpretable handcrafted cellular feature information. Following unsupervised training, the pre-trained weights of the encoder and the projector were used to initialize an image feature extractor for downstream classification of NEPC (Figure 1D and Methods). A multiple-instance learning (MIL) approach was employed to predict labels for cores in the training set (NEPC positive: 149; NEPC negative: 256) using pathologist and IHC positivity (see below) as a reference standard (4). The hyperparameter settings and the training strategies for all steps are discussed in Methods. The final output of the NEURAL-PC model is a single sample-level output score and spatial map probability distribution (heatmap) from all tiles used during MIL inference.

We benchmarked our NEURAL-PC model against other commonly used image feature extractors including ResNet50 (50), Swin Transformer (51) pre-trained on ImageNet, and the UNI (52) foundation model. Additionally, we evaluate a standard CAE model (image only) and a separate model evaluating handcrafted cellular features to understand the relative benefit of the NEURAL-PC model. All models were evaluated using multiple 5-fold cross-validation schemes. The area under the receiver operating characteristic curve (AUROC) and the area under the precision-recall curve (AUPRC) were calculated for each model (Table 1). Interestingly, the use of handcrafted cellular features alone achieved high AUROCs/AUPRCs, outperforming CAE alone, but fusing information during pre-training resulted in the highest performance for NEPC detection (NEURAL-PC AUPRC 0.717, AUROC 0.883). The UNI foundational model closely followed NEURAL-PC with AURPC 0.704 and AUROC 0.900. Given the imbalance in frequency of NEPC and adenocarcinoma, AUPRC is the preferred metric. Other standard models pre-trained from ImageNet (ResNet50, Swin Transformer) had poor AUPRCs, perhaps attributable to domain mismatch and dataset imbalance. It is important to note that NEURAL-PC has a simple structure (see Methods) with much fewer parameters than other transformer-based models, making it efficient to run. When compared with pathologist morphology assessment, median NEURAL-PC score in poorly differentiated carcinomas was 0.214 (0.01–0.91) compared with 0.068 (0.01–0.66) in adenocarcinoma and 0.847 (0.15–0.97) in NEPC morphology (Supplemental Figure 2). Based on the IHC ground-truth panel for NEPC classification, the majority of false negatives were in non-NEPC morphologies (Supplemental Figure 2).

### Independent validation of NEURAL-PC across different cohorts and sample types.

To assess the performance of NEURAL-PC, model prediction scores were first compared with semiquantitative IHC staining for androgen receptor (AR) and 2 well-established and clinically used neuroendocrine markers, synaptophysin (SYP) and insulinoma-associated protein 1 (INSM1) (4), in the UW-RA TMA testing cohorts (Figure 2, A–C). NEURAL-PC scores correlated well with IHC H-scores: high NEURAL-PC probabilities were observed for strong SYP (median 0.832, IQR 0.545–0.865) and INSM1 (median 0.826, IQR 0.504–0.889) staining (H-score ≥ 70), while intermediate H-scores (20 to 70) yielded more variable correlation with SYP (Figure 2B) and INSM1 (Figure 2C). NEURAL-PC spatial prediction maps revealed uniform probability output in cases with consistent AR^+^NE^–^ or AR^–^NE^+^ phenotypes (Figure 3A). In mixed cases, detection was concentrated in spatially distinct regions, reflecting heterogeneous cell states and biomarker expression and demonstrating that the NEURAL-PC can differentiate intratumoral cell state differences.

As a proof of concept to evaluate how well the model is able to discern the transition from an AR-dominant phenotype to an NE-dominant phenotype, we applied NEURAL-PC to a well-established patient-derived xenograft (PDX) model, LTL331, which undergoes reproducible transition from an adenocarcinoma to an AR-independent NEPC following castration (53). Samples from 3 time points representing different stages in the lineage transition cascade were evaluated and compared with IHC as reference standard (Figure 3B). After H&E normalization for mouse tissue, NEURAL-PC prediction maps matched IHC findings across time points. A uniformly negative NEURAL-PC probability map was observed at baseline, with strong AR expression at baseline and negative NEPC expression. In the post-castration setting (week 46), NEURAL-PC showed a uniformly positive probability map within the emergent NEPC tumor with strong NE marker expression and negative AR expression. Importantly, NEURAL-PC correctly identified the emergence of focal NE marker expression during the transitional time point (week 24), with NEURAL-PC spatial probability correctly identifying focal regions of NEPC features. This shows that even in admixed tumors, NEURAL-PC can correctly identify focal NEPC.

To assess the utility of NEURAL-PC in clinical biopsy samples, we evaluated its performance in a retrospective cohort of 63 clinical biopsy samples (UW-MATCH cohort: 24 NEPC, 38 adenocarcinoma, 1 amphicrine). These samples were characterized following published guidelines for IHC confirmation to determine NEPC (4). NEPC cases had higher NE scores (median 0.877, IQR 0.749–0.949) than adenocarcinomas (median 0.243, IQR 0.091–0.523) (Figure 2D). In this cohort, NEURAL-PC achieved an AUROC of 0.921, which was even higher than the mean AUROC reported during cross-validation training in TMA cores (Figure 2E). Using an optimal NE threshold of 0.598 (Youden’s J statistic), the model achieved a sensitivity of 0.875 and positive predictive value of 0.724. Classification performance is summarized in Table 2, with representative prediction maps shown in Figure 3C, highlighting the morphologic consistency in high-attention regions for NEPC samples. NEURAL-PC demonstrated uniformly low probabilities in confirmed adenocarcinoma samples (Figure 3D).

As an additional real-world cohort with limited IHC data, we analyzed 92 slides from an external cohort of 74 patients diagnosed with NEPC-like features, of which 85 slides from 68 patients had sufficient tumor content (Weill Cornell Medical College [WCMC] cohort). Pathology reports were queried to identify all patients with small-cell PC, NEPC, or any overlap of adenocarcinoma and NEPC features consistent with amphicrine carcinomas (3, 22, 54). A summary of pathology report diagnosis and NEURAL-PC classification can be found in Table 3. NEPC validation through IHC was not completed in this cohort. Within these NEPC-like features, NEURAL-PC scores were lower than in IHC-proven cases (median 0.637, IQR 0.245–0.920; Supplemental Figure 3).

To evaluate quality-related impacts on NEURAL-PC performance, we next reviewed low-scoring cases with an expert genitourinary pathologist to further assess performance. In cases in which the cancer detection results in a high number of false-positive regions (normal surrounding tissue), these regions are largely predicted as NE negative by the NEURAL-PC algorithm and therefore result in underestimation of NE proportion and overall prediction in the image (Supplemental Figure 4A). Another notable cause of failures in this cohort were common artifacts in whole-slide images (WSIs) that may also cause detection failure, including scanning artifacts (blur) or tissue artifacts (processing or mounting effects), as shown in Supplemental Figure 4B. Finally, NE detection may fail when there are small/focal NE components in a large sample. The slide shown Supplemental Figure 4C demonstrates that the majority of the slide is represented by typical adenocarcinoma-like features.

### Correlation of NEURAL-PC predictions and tumor transcriptomic features.

To determine the association between NEURAL-PC outputs and tumor transcriptomic features, we leveraged 118 WSIs of large tissue sections from 75 patients for which H&E tissue images as well as bulk RNA-seq data were available (43, 44). Each H&E slide was reviewed by an expert pathologist for detailed morphologic characterization, and NEPC determination was made from a combination of morphology and molecular features. NEURAL-PC’s outputs were well correlated with pathologist-defined morphology group, and NEURAL-PC achieved an AUROC of 0.810 (Supplemental Figure 5).

When the association between NE prediction scores and transcriptomic profiling of tumors was evaluated, specimens predicted as NEPC positive by NEURAL-PC showed high-level expression of NEPC-related genes such as *SYP*, *INSM1*, and *ASCL1*, while specimens predicted as NEPC negative demonstrated higher expression of canonical AR-positive luminal epithelial cells as seen in classic prostate adenocarcinoma such as *KLK3*, *KLK2*, *AR*, *STEAP1*, and *NKX3.1* (Figure 4A). Furthermore, expression of 10 NE-related genes was significantly higher for samples predicted as NEPC positive by NEURAL-PC (*P* = 0.00135; Figure 4B), showing a dominant association with positive expression in NEPC genes and negative expression in AR-related genes (Figure 4C). Correspondingly, there was significantly lower expression of 10 AR-related genes for samples classified as NEPC negative by NEURAL-PC (*P* = 3.92 × 10^–8^; Figure 4D). In addition, in line with the overall higher proliferation rates of NEPC, samples with high NEURAL-PC scores showed higher expression of CCP.31 (Figure 4E), a 31-gene cell cycle progression signature that is tightly correlated with cellular proliferation rates and associated with poor outcomes in PC (55).

### NEURAL-PC predicts outcome in cohorts with aggressive-variant PC.

To assess the relationship between NEURAL-PC–derived predictions and clinical outcomes, we trained a random survival forest (RSF) prognostic model using all model-derived features: slide-level NE prediction, proportion of NE-predicted tiles, and average tile-level probabilities and logits (see Methods) in the UW-MATCH cohort. Out-of-bag predictions from the RSF effectively stratified patients by risk of death within 2 years, with median overall survival of 14.3 months in the high-risk group versus >24 months in the low-risk group (*P* < 0.0001; Figure 5A). This demonstrates that both the overall slide-level likelihood of NEPC and the extent of NEPC features detected by NEURAL-PC within the tumor are prognostic of outcome.

To test this further, we applied the NEURAL-PC prognostic model first to an independent cohort of NEPC, amphicrine, or small-cell PC (WCMC cohort). Among 66 patients with sufficient tumor burden and outcome data, patients predicted as high-risk for death within 2 years had a median survival of 8.62 months versus 19.6 months in those predicted as lower-risk (*P* = 0.075; Figure 5B). While not reaching the statistical significance threshold, this trend suggests that even within NEPC cohorts NEURAL-PC features can provide additional prognostic information.

To further validate the model, we evaluated NEURAL-PC in the C-COLA trial (56, 57), a phase II clinical study (NCT03263650, ClinicalTrials.gov) that assessed the activity of chemotherapy followed by PARP inhibitor maintenance in patients with aggressive-variant PC (19). H&E scans from 82 patients with sufficient tumor burden were evaluated regardless of treatment arm by NEURAL-PC. Here, the NEURAL-PC prognostic model features demonstrated statistically significant association with 2-year outcomes, with the high-risk group demonstrating median survival of 12.3 months versus 20.4 months in patients classified as lower-risk (*P* = 0.01; Figure 5C). The association with outcome was stronger for the RSF model constructed from all NEURAL-PC features (HR 2.27 [95% CI 1.19–4.31; *P* = 0.01]) compared with overall NEURAL-PC classification alone (HR 2.15 [95% CI 0.29–15.8; *P* = 0.45]), demonstrating the prognostic value of incorporating NE prediction burden and heterogeneity across tiles.

## Discussion

Given the biological complexity of metastatic PC, accurate tumor detection and subclassification are of significant clinical importance. The overall objective of this study was to develop a deep learning–based model for accurate detection and quantification of NEPC from H&E slides. To this end, we have introduced an approach, NEURAL-PC, that utilizes so-called handcrafted features of nuclear size, shape, and appearance during training of the deep learning–based CAE to regularize and provide attention to important characteristics of the NEPC phenotype. This methodology outperforms common encoders used in the digital pathology domain and demonstrated significant association with NEPC-related gene expression signatures. NEURAL-PC was trained on diverse specimens reflecting the heterogeneity of metastatic PC and generalized well to multiple validation cohorts spanning PDX to clinical biopsy samples.

Accurate detection of NEPC is critical for effective patient management. However, establishing a definitive diagnosis of NEPC remains challenging in standard pathology practice. Although IHC markers can assist in diagnosis, isolated positivity for a single neuroendocrine marker, for example, can be misinterpreted as evidence of NEPC, leading to tumor misclassification and potentially inappropriate or excessive treatment (4). Conversely, failure to recognize NEPC — or delays in diagnosis — can prevent patients from receiving timely, aggressive therapy for this rapidly progressive disease.

These challenges highlight the urgent clinical need for robust diagnostic tools that can be applied broadly to all metastatic PC biopsies to evaluate the presence and extent of NEPC. Notably, our studies demonstrate that the NEURAL-PC assay can detect even small clusters of emerging NEPC, underscoring its potential utility for identifying even early or incipient NEPC lesions.

In addition to its diagnostic capability, we demonstrate that NEURAL-PC provides prognostic information, enabling the stratification of clinically aggressive variants of PC further into prognostic subgroups. By combining overall classification results with other tumor metrics including the proportion of cancer predicted as NEPC, and the spatial distribution of model outputs, NEURAL-PC can separate patients into prognostic groups. This enhanced prognostic value suggests that the heterogeneity in morphologic patterns contains clinically relevant information. Broadly, this observation aligns with prior reports highlighting the phenotypic heterogeneity of NEPC (20). For instance, tumors with “mixed” histologies have been shown to have more favorable outcomes compared with pure NEPC (3, 19).

While deep learning excels at capturing complex and hierarchical histologic patterns, such models are often criticized for their lack of interpretability. In contrast, handcrafted cellular features — based on established biological knowledge — have demonstrated prognostic value across multiple cancer types (49, 58–60). These features offer interpretability and can be engineered for robustness to technical variation, enhancing generalizability.

Prior studies have explored feature fusion strategies (61–63), typically treating deep and handcrafted features as independent inputs fused by concatenation or attention mechanisms. Our approach differs by integrating cellular features directly into the feature extraction process. Specifically, we regularize a CAE by projecting the encoder’s output into a fixed latent space for cellular feature reconstruction. This hybrid strategy improves both image reconstruction and model interpretability. Notably, our model requires only a single feature extraction backbone at inference time, thereby eliminating the need for separate handcrafted feature computation while retaining the benefits of interpretable guidance during training. The same architecture holds potential for multimodal fusion, transfer learning, and knowledge distillation in future applications.

NEURAL-PC was trained and validated across a wide range of clinically relevant specimens including samples from different metastatic sites, specimen types, and biopsies obtained for a variety of clinical indications. Despite this diversity, we observed stable and reproducible performance, demonstrating the robustness of NEURAL-PC. To achieve this, we implemented a cancer detection module to preselect tumor regions, reducing bias from varying metastatic sites and surrounding tissue microenvironments. This ensured sample type–independent performance. Further, our user-in-the-loop workflow and interpretable outputs also allow for refinement of annotations by expert pathologists when necessary. Nonetheless, our end-to-end evaluation revealed that rare NEPC misclassifications stemmed from tumor detection errors and tissue-related crush artifacts. Further evaluation of slide-related tissue artifacts and quality issues including image blur are an area for future improvement.

This study has several limitations. While model development was performed on a large number of tissue samples, these ultimately came from a relatively small cohort of rapid autopsy patients, which may not be fully representative of all disease phenotypes. However, careful validation in 3 settings (PDX, clinical biopsy, and external clinical samples) was performed, including an external site and digital scanners. Future prospective work and validation at additional clinical sites are warranted. Incomplete records or inter-reader variability may have an impact on results. Ground-truth NEPC determination was made from a panel of IHC markers in the training set. Discrepancies between NEPC-related IHC markers and model performance were not assessed owing to small incidence in the test set. Similarly, a small set of amphicrine tumors were available for rigorous evaluation. The impact of variable IHC expression and amphicrine-like tumors is a topic for future consideration. Our model was challenged against several state-of-the-art encoders for digital pathology; however, as foundation models continue to emerge, future evaluation is warranted. In addition, future studies are warranted to evaluate the correlation of our NEURAL-PC model with clinical features of prognostic relevance and its use as a predictive biomarker in various treatment settings. 

In summary, we developed a convolutional autoencoder regularized by handcrafted nuclear features, NEURAL-PC, to enable robust detection and characterization of NEPC on routine H&E-stained slides. By leveraging cellular morphology, the NEURAL-PC model outperformed multiple state-of-the-art classifiers, including foundation models. It demonstrated strong generalizability across diverse validation cohorts, including clinical biopsies. Given the molecular heterogeneity of late-stage prostate cancer and reliance on IHC for NEPC diagnosis, this H&E-based AI model offers substantial potential to enhance diagnostic precision and streamline pathology workflows.

## Methods

### Sex as a biological variable

All subjects in this research were biologically male, since the prostate as an organ and prostate cancer as an entity exist in only males.

### Experimental model and study participant details

#### University of Washington rapid autopsy cohort.

All rapid autopsy tissues were collected from patients enrolled in the Prostate Cancer Donor Program at the University of Washington. Formalin-fixed, paraffin-embedded tissues from various metastatic sites in 52 patients were used to construct tissue microarrays (TMAs), as described previously (43, 44). IHC of various molecular targets was available from prior published studies (45–47). For all IHC assays, a semiquantitative measure of staining level was reported, H-score, with a range of 0–200 (47). Transcriptional profiling was completed for a subset of tumors within this cohort, as described previously (24) and available through the NCBI’s Gene Expression Omnibus database (GSE147250, GSE228283). Corresponding H&E images from tumor sections undergoing transcriptional analysis were collected. All slides were digitized using a Ventana DP 200 Slide Scanner (Roche) at 0.25 μm/pixel (effective ×40 magnification), and deidentified images were evaluated as part of this study.

#### University of Washington biopsy cohort.

A retrospective cohort of 63 patients with metastatic PC undergoing routine clinical metastatic biopsies at the University of Washington were identified after a review of pathological and clinical data. IHC studies, when performed as part of routine diagnostic workup, were included in the evaluation. Samples were evaluated for the presence of neuroendocrine-like features, based on morphologic and/or molecular features, by an expert genitourinary pathologist (4). All slides were digitized using a Ventana DP 200 Slide Scanner (Roche) at 0.25 μm/pixel (effective ×40 magnification), and deidentified images were evaluated as part of this study. Full clinical history including initial diagnosis, all prior treatments, molecular assays, and clinical follow-up was collected. Specifically, outcomes related to metastatic PC, including date and cause of death or last follow-up, were collected.

#### Weill Cornell Medical Center cohort.

A retrospective cohort of 74 patients with metastatic PC who were clinically identified as having neuroendocrine-like differentiation, and any specimens obtained throughout the course of care (biopsy) or death (autopsy) were reviewed. All slides were digitized using an Aperio scanner (Leica) at 0.25 μm/pixel (effective ×40 magnification), and deidentified images were evaluated as part of this study. Clinical history including diagnosis and treatment of PC, emergence of neuroendocrine phenotype, and date from biopsy to death or last follow-up was collected.

#### MD Anderson Cancer Center cohort.

A clinical trial cohort of 82 patients with metastatic PC enrolled in the C-COLA trial (56, 57), a phase II clinical study that assessed the activity of chemotherapy followed by PARP inhibitor maintenance in patients with aggressive-variant PC were reviewed (19). All slides were digitized using an Aperio scanner at 0.5 μm/pixel (effective ×20 magnification), and deidentified images were evaluated as part of this study. Image tiles were resampled to ×40 magnification for analysis. Response to therapy and date from biopsy to death or last follow-up were collected per protocol.

#### LTL331 PDX model.

Cryopreserved pieces of LTL331 PDX (53) were engrafted into the subrenal capsules of intact immunodeficient host mice (NOD.Cg-*Prkdc^scid^* *Il2rg^tm1Wjl^*/SzJ, JAX 005557, The Jackson Laboratory). When tumors reached approximately 150–200 mm^3^, as determined by MRI, they were excised, and a portion was implanted subcutaneously (SQ) into the flanks of recipient mice and designated LTL331 SQ UBC. For this experiment, LTL331 SQ UBC tumors were implanted subcutaneously into intact male mice and allowed to grow for approximately 2 months followed by castration and tumor resection at specific time points post-castration. Tumors were harvested at the assigned post-castration time point or when they reached the maximum allowable size of approximately 2,000 mm^3^. Portions of the tumors were fixed in 10% neutral-buffered formaldehyde, transferred to 70% ethanol, and sent to the Fred Hutch Experimental Histopathology Core for paraffin processing.

Formalin-fixed, paraffin-processed tumors were embedded into composite paraffin blocks, sectioned at 4 μm, and mounted on positively charged slides. Sections were baked at 65°C for 1 hour to remove excess paraffin, then loaded onto the Ventana Discovery Ultra automated staining platform (Ventana Medical Systems). Slides underwent onboard deparaffinization in Discovery Wash Buffer (Roche Diagnostics, 950-510), followed by heat-induced epitope retrieval using Discovery CC1 solution (Roche Diagnostics, 950-224). Sections were stained with primary antibodies against AR (rabbit, D6F11, 1:100; Cell Signaling Technologies), INSM1 (mouse, A8, 1:100; Santa Cruz Biotechnology), or SYP (rabbit, SP11, 1:100; Genetex) diluted in Ventana Antibody Diluent with Casein (Roche Diagnostics, 760-219). For INSM1 staining, slides were further incubated with a rabbit anti-mouse IgG secondary antibody (1:400; Abcam) before detection. Detection was carried out using the DISCOVERY anti–rabbit HQ secondary antibody (Roche Diagnostics, 760-4815) and anti-HQ–HRP enzyme conjugate (Roche Diagnostics, 760-4820), followed by visualization with ChromoMap DAB (Roche Diagnostics, 760-159). Sections were counterstained with Hematoxylin II (Roche Diagnostics, 790-2208) and Bluing Reagent (Roche Diagnostics, 760-2037), then scanned at ×40 magnification using the Ventana DP 200 digital slide scanner.

### Image processing

All WSIs underwent evaluation by a cancer detection algorithm to limit the influence of non-tumor-associated pathology features, such as site of metastasis and surrounding microenvironment. Briefly, the cancer detection algorithm was trained from 165 WSIs from metastatic biopsy (*n* = 95), TMA core (*n* = 58), and normal tissue images (*n* = 12; lymph node/adrenal controls). Initially, a subset of images were annotated by 2 pathologists using QuPath software (https://qupath.github.io). A DeepLabV3 segmentation model (https://github.com/WaterKnight1998/SemTorch) was trained at ×20 magnification with patch size 250 × 250. As visually depicted in Figure 1A, the model was iteratively applied to new WSIs, converted to QuPath-friendly format (64) manually adjusted by pathologists, and retrained from adjusted annotations until no major refinements were needed, using user-in-the-loop process defined previously (65). The final model achieved a Dice score of 0.761 in a validation set. This model was then applied to all images in the study and was used to select tiles with more than 10% cancer area of size 250 × 250 at ×40 magnification. Tiles with more than 45% white space were also discarded, resulting in 247,203 tiles (215,104 for training and 32,099 for validation). Cellpose (48) was used to segment nuclei and extract a cellular feature vector (49) for each tile. All cellular features were normalized by *z* score transformation using mean and standard deviation calculated across all training TMA cores.

### Stain normalization

For all experiments with an external validation dataset, we applied the Macenko stain normalization (66) to ensure the consistency of the model inference. Specifically, the Macenko method estimates the stain vectors of a given tile image using singular value decomposition. Then these stain vectors were normalized to some given references, and these new stain vectors were used to construct the normalized images. The code we used in our experiments for stain normalization is available at https://github.com/zjchen10/stainnorm_pytorch

### NEURAL-PC model architecture

The proposed NEURAL-PC model consists of an encoder *f*, a feature projection module *p*, an image decoder *g_I_*, a cellular feature decoder *g_C_*, and a classifier module *h*. Briefly, each tile was first transformed by an encoder *f* and a projector *p* into 2 latent vectors *z_m_* and *z_c_*. The image decoder *g_I_* uses the sum *z* = *z_m_* + *z_c_* to reconstruct the input tile image for regularization, while the feature decoder *g_C_* uses only *z_c_* to reconstruct the cellular feature vector.

#### Encoder f.

Illustration of *f* is shown in Supplemental Figure 1A. It is a convolutional network that consists of several residual blocks, where the overall architecture of *f* is similar to ResNet34 (Supplemental Figure 1B). To better preserve the spatial information in the image, we replace the pooling operation with stride 2 convolution. Let 
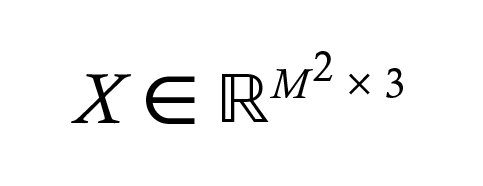
 be an input image of size *M*; the encoder *f* transforms it into a 3D pre-feature map 
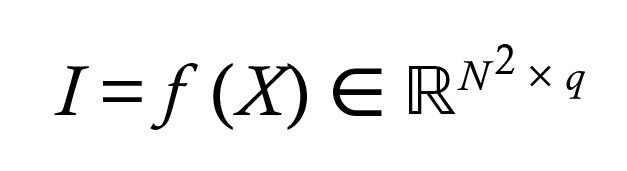
.

#### Feature projector p.

This module projects the pre-feature map *I* into a low-dimensional latent space and splits the projection into 2 parts. A linear layer followed by nonlinear tanh activation first transforms the pre-feature map into a coefficient vector 
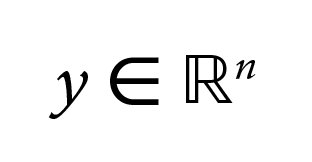
; then the image feature *z* is obtained by matrix-vector product *z* = *Dy*, where 
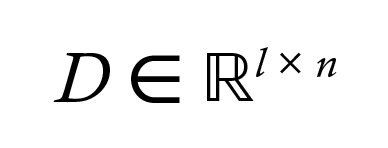
 is a fixed and non-trainable dictionary matrix whose columns are independent and identically distributed (i.i.d.) sampled from uniform distribution over the unit sphere (40). This design allows us to decompose *z* into 2 “almost orthogonal” vectors. Specifically, if we view *y* as the concatenation of 2 vectors 
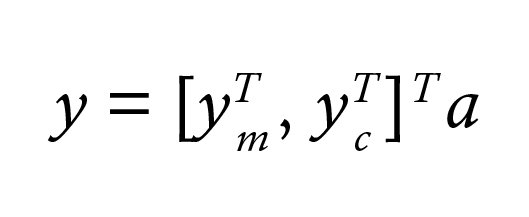
and *D* as the concatenation of 2 submatrices *D* = [*Dm*, *Dc*], then

*z* = *Dy* = *D_m_y_m_* + *D_c_y_c_* = *z_m_* + *z_c_*

Since columns of *D* are i.i.d. random vectors, the inner product 
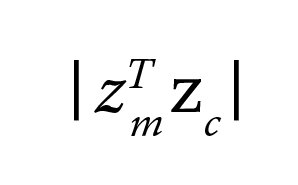
 is close to 0 with high probability. *z_m_* is referred to as the main part, which contains general visual information, and *z_c_* is referred to as the complementary part, which will be used to reconstruct cellular features. The process is shown in Supplemental Figure 1C.

#### Image decoder g_I_ and cellular feature decoder g_C_.

These 2 decoder networks use features from *p* to reconstruct images and corresponding cellular features. *g_I_* is a deconvolutional network whose structure is symmetric to that of the encoder *f*, while *g_C_* is a simple feedforward network. For image reconstruction, *g_I_* uses the feature *z*. For cellular feature reconstruction, *g_C_* uses the complementary *z_c_*, i.e., only a part of the image feature is used. Illustration of *g_I_* is shown in Figure 1C.

#### Classifier h.

For downstream supervised classification tasks, we connect a classifier module *h* to the feature projector *p* (Figure 1D). Since we only have weak label for the cores/slides, we adopt the multiple-instance learning (MIL) scheme. The module *h* contains an attention block (67) that congregates a collection of image features into a single output. Let *Z* = [*z*_1_, …, *z_L_*]*^T^* be a bag (or a collection) of all tile features from one core/slide image; the classifier *h* outputs the core-level feature as follows:

*Y* = *MultiHeadAttention*(*Q*,*K*,*V*)

where 
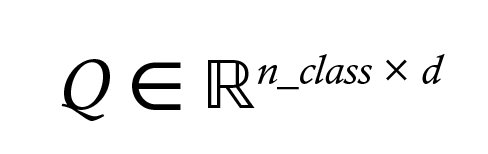
 contains trainable class tokens as its rows, *K* = *W^K^Z* and *V* = *W^V^Z*. Note that this attention block can take any number of tile features. A feedforward neural network with sigmoid activation then takes this core/slide-level feature *Y* and outputs a probability/score. The architecture of *h* is shown in Supplemental Figure 1D.

The sizes of the pre-feature map *I*, coefficient vector *y_m_*, coefficient vector *y_c_*, and feature vector *z* were set to 16 × 16 × 16, 1,600, 300, and 1,600, respectively, in our experiments.

### Loss functions

During the pre-training phase, we train *f*, *g_I_*, and *g_C_* for image and cellular feature reconstruction. The following loss terms are used in this stage.

#### Image MSE loss.

Image mean squared error (MSE) loss is defined as the mean-square difference between the input image *X* and the reconstructed image *X̃*,
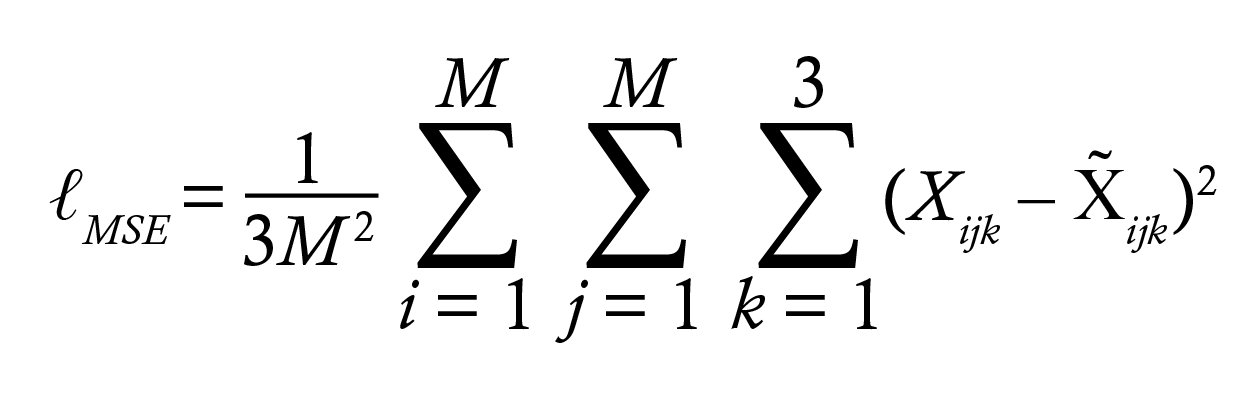


where *X_ijk_* is the value of the *k*-th channel of pixel at position (*i*, *j*) and *M* is the size of the image.

#### Image SSIM loss.

Optimizing *ℓ_MSE_* alone will produce blurry reconstructed images and cause structural information loss. To better preserve this information, we use the structural similarity index measure (SSIM) loss *ℓ_SSIM_*. The SSIM between 2 images is defined as *SSIM* (*X*, *X̃*) = *l* (*X*, *X̃*) × *c* (*X*, *X*) × *s* (*X*, *X̃*), where *l*, *c*, and *s* are luminance, contrast, and structural comparison functions, respectively. The SSIM loss is then defined as

*ℓ_SSIM_ =* 1 *– SSIM* (*X*, *X̃*)

#### Cellular feature MSE loss.

Let *c* be the cellular feature vector that corresponds to the input image, and *c̃* = g_c_ (z_c_) be the reconstructed cellular feature vector. Then the cellular feature MSE loss *ℓ_CMSE_* is defined as the mean-square difference between *c* and *c̃*:
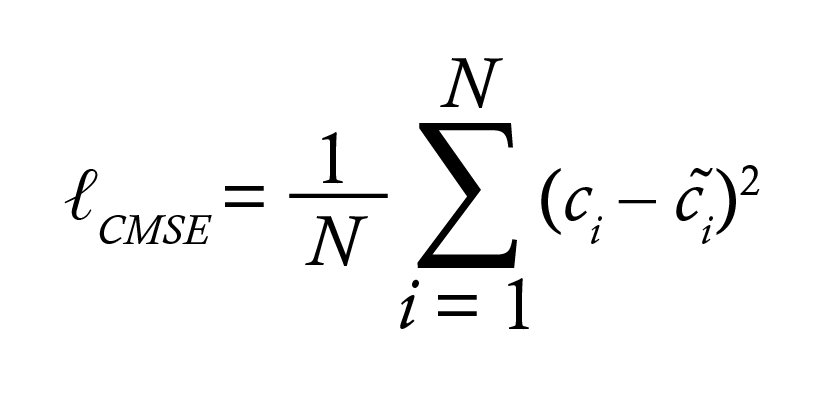


The loss function we minimize is the weighted sum *ℓ* *=* λ_1_ × *ℓ_MSE_ +* λ_2_ × *ℓ_SSIM_* λ_3_ × *ℓ_CMSE_*. The weights used in our experiment were λ_1_ = 1, λ_2_ = 0.3, and λ_3_ = 1.

For the downstream NEPC classification task training, the binary cross-entropy is used as the loss function. Note that only parameters of the projector *p* and the MIL module *h* are updated at this step.

### Multiple-instance learning framework

Here the encoder is fixed but the weights of the projector will be optimized. The encoder and the projector first transformed the bag of tile images into the bag of tile features. An attention-based MIL module then congregated the bag of tile features and output prediction for the input core/slide (Figure 1). Binary cross-entropy is used as the loss function for training.

### Reference standard label for NEPC

The reference gold standard for determining NEPC status was based on IHC criteria from Haffner et al. (4). Briefly, NEPC markers from synaptophysin (SYP) and insulinoma-associated protein 1 (INSM1) were used in combination with androgen receptor (AR) pathway/prostate lineage markers from AR and NKX3.1. Following the expert criteria, each marker was scored for the percentage of positive cancer cells and overall intensity. IHC markers were semiquantitatively evaluated by H-score, reflecting the positivity of protein expression in a sample. H-score greater than 20 was considered positive as described previously (7). In rare cases in which initial origin of metastasis was unknown, prostatic origin was confirmed by IHC for HOXB13 (68). Samples with morphology reflecting a suspected secondary or non-prostate origin were excluded. These molecular markers are reported in addition to morphology classification into the following criteria: small-cell carcinoma, adenocarcinoma, poorly differentiated carcinoma, and other. These gold standard labels were available in training (UW-RA) and biopsy testing (UW-MATCH) cohorts.

### Visualizing model performance via prediction heatmap

To visualize the model’s performance, we computed and stored the un-normalized scores (logits) for all individual tiles extracted from the core/slide by applying the classifier head to those tile features. These individual tile scores were then mapped linearly onto the indices in the colormap RdBu_r with the center set to be 0, and spatially aggregated to form the detection heatmap for the input core/slide. Tiles with positive predictions (positive scores, contribute to positive prediction of the input core/slide) were displayed in red, and tiles with negative predictions (negative scores, contribute to negative prediction of the input core/slide) were displayed in blue.

### Experimental setup

For semi-supervised pre-training, modules *f*, *p*, *g_I_*, and *g_C_* were trained by optimization of the loss function *ℓ* with the Adam (69) optimizer using learning rate 1 × 10^–4^, exponential decay rate 0.975, and batch size 100. Data augmentation such as random flip and rotations was used. We also randomly corrupted half of the images with Gaussian noise (mean 0, variance 0.04) during training. The model was trained for a maximum of 50 epochs. The training was stopped when the image MSE loss *ℓ_MSE_* < 0.008, *ℓ_SSIM_* < 0.4, and *ℓ_CMSE_* < 0.3.

For NEPC classification training on the rapid autopsy TMA cohort, we used multiple (*n* = 5) 5-fold cross-validation (split at patient level) to evaluate the performance of each model. All models were trained for a maximum of 40 epochs with the Adam optimizer using learning rate 1 × 10^–5^, exponential decay rate 0.95, and batch size 1. Training was stopped when validation did not decrease for 5 epochs in a row, and weights that achieved the lowest validation error were stored for performance evaluation.

All experiments were performed on the NIH High-Performance Computing (HPC) Biowulf cluster (https://hpc.nih.gov/).

### Random survival forest

To evaluate the clinical utility of the NE prediction model in biopsy samples, all model output features were considered for input to a random survival forest model to predict time to death. Variables considered for model input included overall slide-level NE prediction (value 0–1) from NEURAL-PC, total number of tiles from the sample, proportion of individual tiles predicted as NE positive by NEURAL-PC, proportion of individual tiles predicted as NE negative by NEURAL-PC, average model logits from all tiles, average model attention from all tiles, and average model prediction probability from all tiles. The individual tile-level predictions were determined through the same process as described above for visualization and heatmap generation. The model was trained from the UW-MATCH cohort, with outcomes censored to death within 2 years, with the Fast Unified Random Forests for Survival, Regression, and Classification (RF-SRC) package in R using log-rank splitting during model fit (70). Out-of-bag predictions were reported for the training set, and the model was applied to the WCMC cohort as external validation using the survex (Explainable Machine Learning in Survival Analysis) library (71).

### Statistics

The main evaluation metrics for NEPC classification were the area under the receiver operating characteristic curve (AUROC) and the area under the precision-recall curve (AUPRC). The threshold that maximized Youden’s J statistic was chosen as the threshold for NEPC classification. Sensitivity and positive predictive value were calculated for NEPC classification performance evaluation. For predicted NE score distributions, we presented the median as well as the interquartile range (IQR). Statistical evaluation of NEURAL-PC classification compared with gene expression was completed using Wilcoxon’s rank sum test. These statistical analyses were conducted using Python (version 3.10), PyTorch (version 1.12.1), Scikit-learn (version 1.3.0), Seaborn (version 0.12.2), and R (version 4.3.3).

### Study approval

The study was approved by the University of Washington (IRB 2341) and Fred Hutchinson Cancer Center (IRB 10706) Institutional Review Boards in Seattle, Washington, USA. All participating men provided written informed consent for a rapid research autopsy and tissue procurement. The Institutional Review Board of WCMC in New York, New York, USA, approved this study (IRB 20-06022185), and subjects provided informed consent. The Institutional Review Board of MD Anderson Cancer Center in Houston, Texas, USA, approved this study (IRB 2017-0133), and subjects provided informed consent (Clinical trial number: NCT03263650). This research was approved by the NIH in Bethesda, Maryland, USA (IRB 001088); consent was not required due to its determination as non-human-subjects research involving only anonymized images.

### Data availability

All values for graphical figures are summarized and available in the Supporting Data Values file. Links to data are provided within the article or its supplemental information files, when applicable. Code used for stain normalization is available at https://github.com/zjchen10/stainnorm_pytorch; commit ID 24c50d6522491c1f73ff3924a33f31795c566b2e. Code used to train and generate model predictions and probability maps is available at https://github.com/NIH-MIP/NEURAL-PC; commit ID 85134b37cc1580172ed973d89de33da1ca373179, and model weights are available upon request. H&E WSI imaging data are not publicly available; readers are encouraged to contact the corresponding author(s) for reasonable requests.

## Author contributions

ZC, ES, HR, HZ, LL, PC, BT, MCH, and SAH contributed to model development, design, computational resources, performance review, and validation. MTS, RR, LG, EYY, HHC, JTN, AM, DN, ST, HPB, PSN, AA, CNS, and MCH contributed to patient accrual. ES, DG, RP, AG, IC, RD, CM, YW, CCV, CF, JO, TK, JM, MS, PSN, RS, CNS, and MCH contributed to data collection, including clinical information and/or clinical specimens. ES, CKCD, JMM, and MCH contributed to pathological review. RD, YW, and PSN contributed to PDX generation and/or tissue preparation. ZC, HR, RE, MCH, and SAH contributed to statistical analysis. ZC, IC, RD, CM, MTS, EYY, HHC, CKCD, HPB, PSN, AA, CNS, MCH, and SAH contributed to manuscript writing. All authors reviewed the manuscript.

## Conflict of interest

CKCD received research funding support from Intuitive Surgical. CNS has served as consultant/advisory board member for Astellas Pharma, AstraZeneca, Bayer, Bristol Myers Squibb/Medarex, Gilead, Merck, MSD, Pfizer, Janssen, Roche, UroToday, OncoLive, Duality Biologics, and Tolmar. HHC received research funds to University of Washington from Clovis Oncology, Color Genomics, Janssen, Medivation, Promontory Pharmaceutics, and Sanofi. MTS received research funding support from Novartis, Zenith Epigenetics, Eli Lilly, Bristol Myers Squibb, Merck, Immunomedics, Tmunity, SignalOne Bio, Epigenetix, Xencor, Incyte, Ambrx, Oric Pharmaceuticals, AstraZeneca, Jenssen, and Pfizer and serves on advisory boards for Sanofi, Fibrogen, Daiichi Sankyo, Janssen, and Pfizer. EYY received research funding support (University of Washington) from Dendreon, Merck, SeaGen/Pfizer, Blue Easter, Bayer, Lantheus, and Tyra and has performed consulting for Astellas, Johnson & Johnson, AstraZeneca, Tolmar, Merck, Bayer, Lantheus, Bristol Myers Squibb, and Loxo. MCH served as a paid consultant for or received honoraria from Pfizer and AstraZeneca and has received research funding from Merck, Novartis, Genentech, Promicell, and Bristol Myers Squibb.

## Funding support

This work is the result of NIH funding, in whole or in part, and is subject to the NIH Public Access Policy. Through acceptance of this federal funding, the NIH has been given a right to make the work publicly available in PubMed Central.

- Center for Cancer Research, National Cancer Institute (NCI), National Institutes of Health (NIH) Intramural Research Program project ZIABC012163 (to SAH).
- NIH/NCI 1P01CA298991-01 to MCH and PSN.
- NIH/NCI R37CA286450 to MCH.
- NIH/NCI P30CA15704 (Fred Hutch/University of Washington Cancer Center Support Grant) to PSN (Cancer Center leadership).
- NIH/NCI P50CA097186 (Pacific Northwest Prostate Cancer SPORE) to PSN and HHC.
- NIH/NCI P01CA163227 to PSN and MCH.
- NIH/NCI R01CA234715 and R01CA266452 to HPB.
- NIH/NCI R01CA280056 to MCH.
- NIH Office of Research Infrastructure Programs S10OD028685 to IC.
- US Department of Defense Prostate Cancer Research Program W81XWH-20-1-0111 to PSN; W81XWH-21-1-0229 to HHC; W81XWH-22-1-0278/W81XWH-22-1-0279 to MCH and SAH; W81XWH-18-1-0347 to PSN; W81XWH-18-1-0689 to HHC; W81XWH-21-1-0264 to MTS; W81XWH-14-2-0183 to PSN; W81XWH-17-2-0043 to HHC; PC230420 to MCH; and PC230582 to HHC.
- Doris Duke Charitable Foundation grant 2021184 to HHC.
- V Foundation grant to MCH.
- PCF Felix Feng PC-SYNERGY Award to PSN and MCH.
- Safeway Foundation grant to MCH.
- Richard M. Lucas Foundation grant to PSN.
- Brotman Baty Institute for Precision Medicine grant to MCH.
- University of Washington/Fred Hutchinson Cancer Center Institute for Prostate Cancer Research grant to MCH.

## Supplementary Material

Supplemental data

Supporting data values

## Figures and Tables

**Figure 1:**
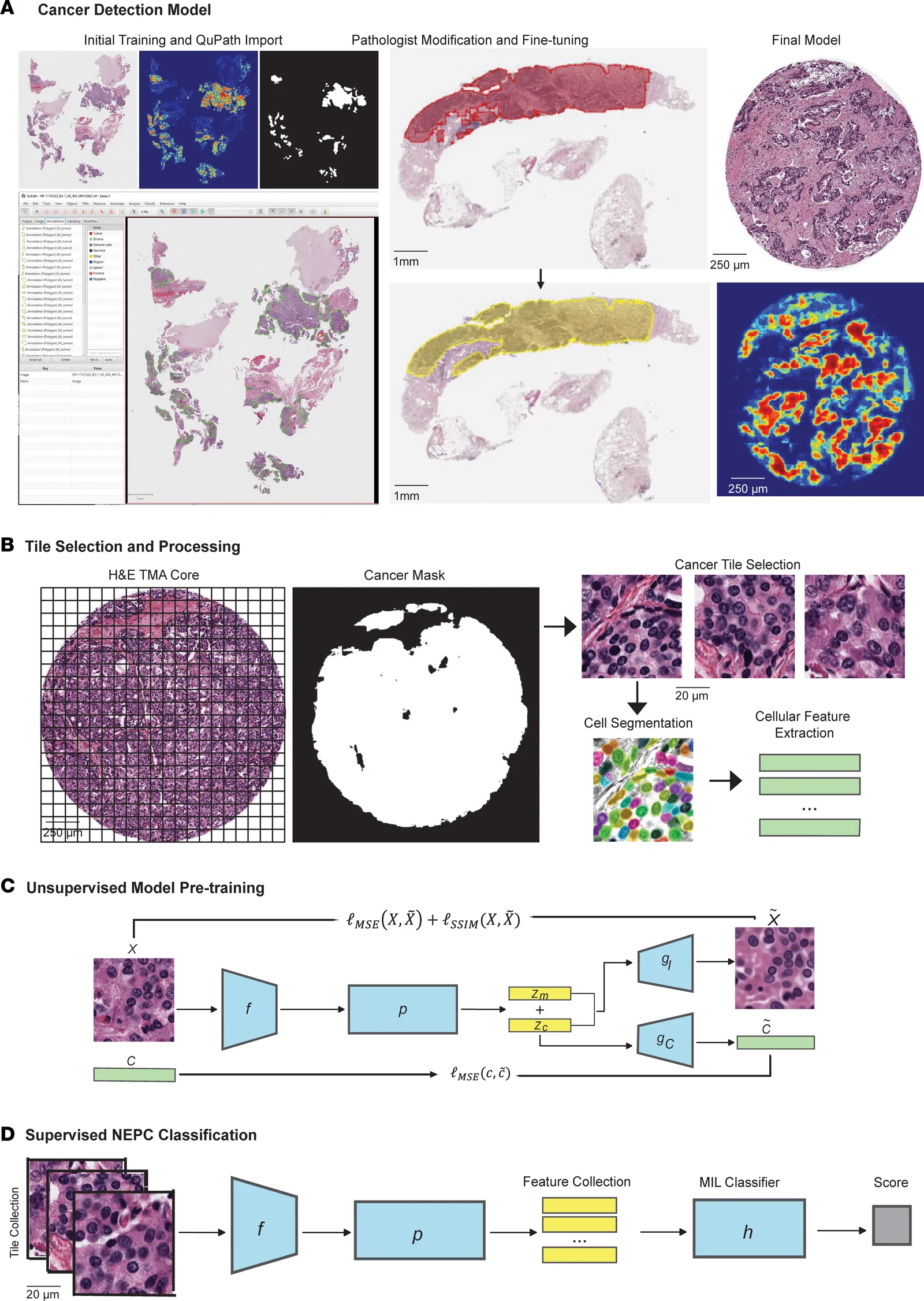
The workflow of the proposed NEURAL-PC model. (**A**) Following initial training from a subset of manually annotated images, a preliminary cancer detection algorithm was applied to each WSI, resulting in a probability map output and binary segmentation mask, which was then converted to GeoJSON format for import to QuPath and manual refinement by pathologists. General modifications to reduce false positives were then used for retraining the cancer detection model. The final model results in highly specific segmentation of cancerous regions, which performs well on various tissue sources. (**B**) Tiles of size 250 × 250 at ×40 magnification with more than 10% cancer area were selected, nuclei were segmented using the Cellpose (48) algorithm, and the cellular feature (59) vectors corresponding to these tiles were extracted. (**C**) In the unsupervised pre-training step, the model was trained to reconstruct tiles as well as the cellular features. Tiles are transformed by an encoder, *f*, and a projector, *p*, into latent vectors *z_m_* and *z_c_*; then a decoder, *g_C_*, uses only *z_c_* to reconstruct the cellular feature vector corresponding to the input tile, and another decoder, *g_I_*, uses the sum *z* = *z_m_* + *z_c_* to reconstruct the input tile image. The objective is to minimize the differences between the input tile image and the reconstructed tile image, as well as the difference between the input cellular features and the reconstructed cellular features (see Methods). (**D**) The trained encoder *f* and projector *p* were combined with a multiple-instance learning classifier *h* to aggregate all tile features corresponding to an input image to produce a core/slide-level NEPC prediction. Digital WSIs were originally acquired at 0.25 μm/pixel (×40 magnification); scale bars are shown for each image.

**Figure 2:**
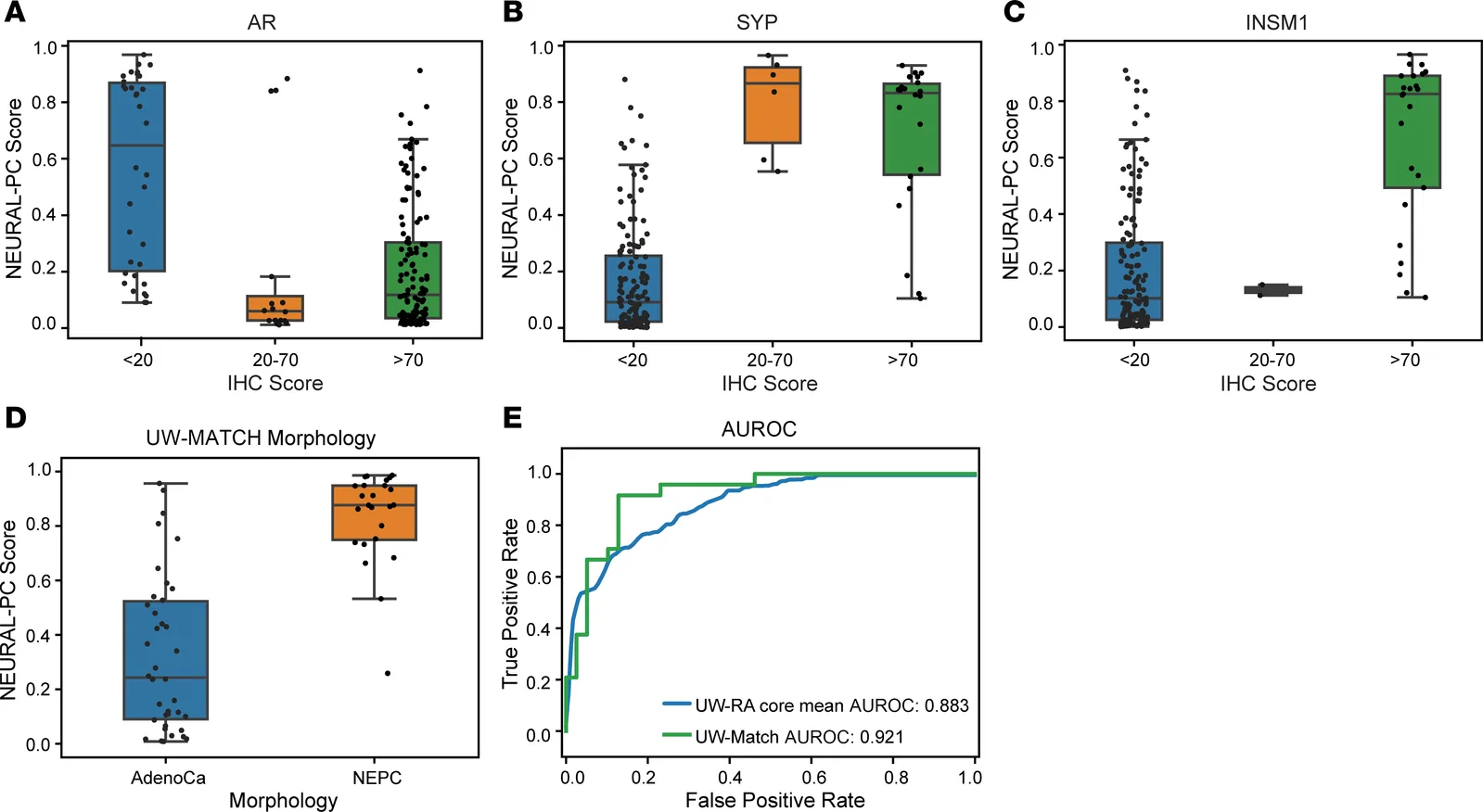
Model performances on internal and external validation cohorts. (**A**–**C**) NEURAL-PC predicted NE score distributions for UW-RA TMA testing cores in different IHC score groupings for AR (**A**), SYP (**B**), and INSM1 (**C**). Generally, ≤20 is considered low/weak expression, 20–70 moderate expression, and ≥70 strong expression. (**D**) NEURAL-PC predicted NE score distributions for UW-MATCH cohort. AdenoCa, adenocarcinoma; NEPC, neuroendocrine prostate cancer with strict IHC guideline adherence. (**E**) AUROC curves for UW-RA TMA core dataset (mean AUROC from cross-validation) and UW-MATCH WSI dataset, respectively.

**Figure 3:**
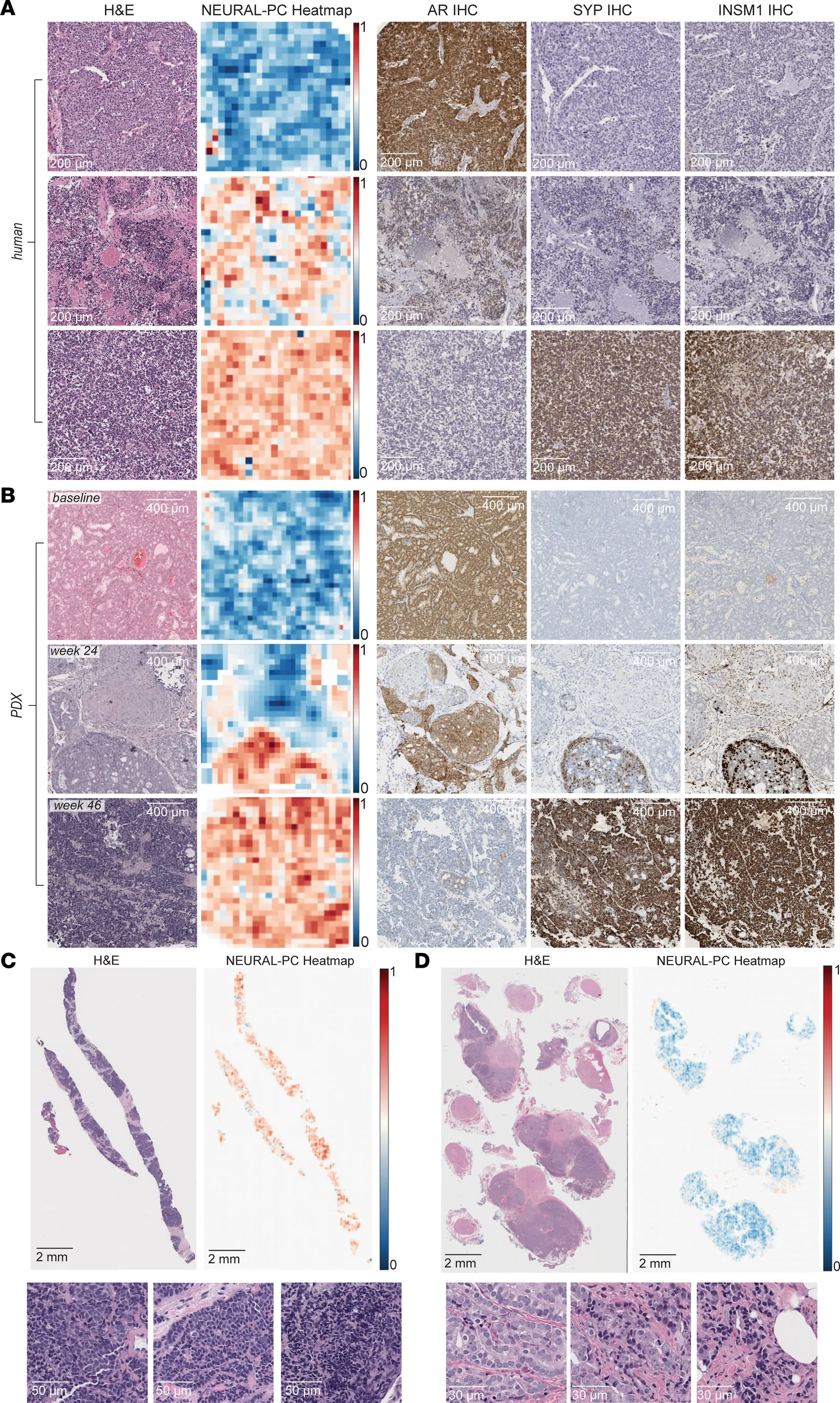
Representative visual NEURAL-PC predictions and spatial IHC association. (**A**) Representative test core images from UW-RA TMA set. High-power view of H&E morphology, NEURAL-PC probability maps, and corresponding AR, SYP, and INSM1 IHC stains. Top: A true negative from AR positive (AR H-score: 160; SYP H-score: 0; INSM1 H-score: 10) with NEURAL-PC prediction < 0.01 and uniform negative/low heatmap. Middle: A mixed-phenotype true positive (AR H-score: 40; SYP H-score: 40; INSM1 H-score: 0) with NEURAL-PC prediction 0.83 and focal positivity in prediction heatmap. Bottom: A true positive from NE positive (AR H-score: 0; SYP H-score: 200; INSM1 H-score: 150) with NEURAL-PC prediction 0.93 and uniform positive prediction heatmap. (**B**) Representative images from PDX validation set. NE prediction heatmaps from NEURAL-PC model are shown separately, along with AR, SYP, and INSM1 IHC stains. Top: AR-positive sample from baseline/untreated PC, correctly predicted as negative by NEURAL-PC (probability score 0.04) with uniform low probability. Middle: Mixed phenotype from transitionary phase (week 24), predicted as negative by NEURAL-PC (0.154) with focal AR and NE IHC marker positivity. Bottom: NE-positive sample from post-castration (week 46) PDX model, correctly predicted as positive by NEURAL-PC (probability score 0.851) with uniform positive prediction map. (**C**) Representative true-positive classification from UW-MATCH cohort. NEURAL-PC probability map reflects uniformly positive predictions across the tissue sample, with overall slide classification score 0.948. Homogeneous morphology is observed across the sample (tile regions shown below). (**D**) Representative true-negative classification from UW-MATCH cohort, with overall NEURAL-PC classification score of 0.055. The majority of the cancer burden shows adenocarcinoma-like morphology (left and middle tiles). Notably, a small proportion of focal areas demonstrate higher tile-level probabilities for containing NEPC in the NEURAL-PC heatmap (right tile region). Digital WSIs were originally acquired at 0.25 μm/pixel (×40 magnification); scale bars are shown for each image.

**Figure 4:**
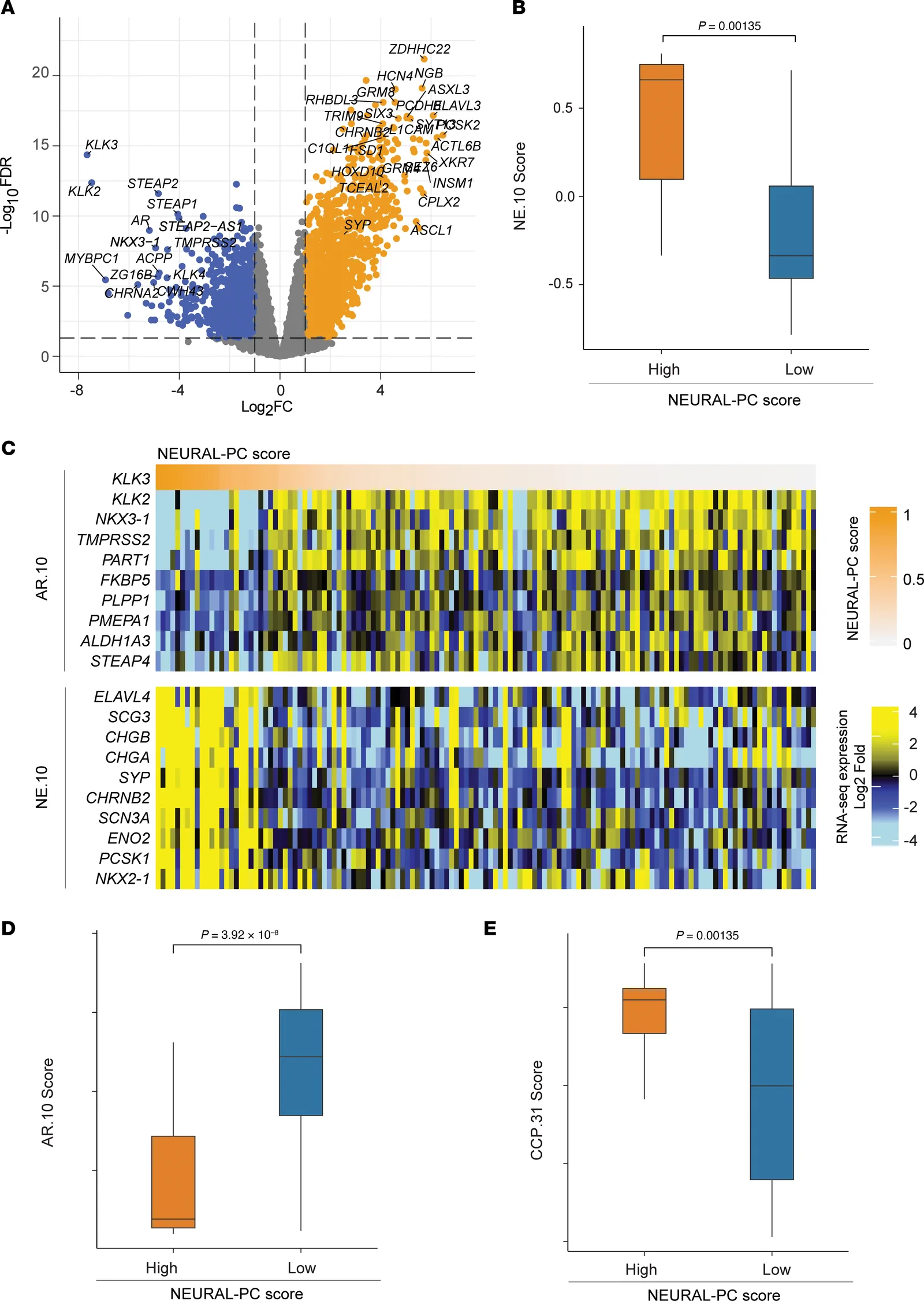
Association with RNA-seq expression in UW-RA WSI cohort. (**A**) Differential expression analysis for WSIs called as positive for NEPC by NEURAL-PC algorithm (upregulated on right) versus those called as negative for NEPC. (**B**) NE.10 expression score versus NEURAL-PC binary classification. (**C**) Association with gene expression profiles in 10 NE-related genes and 10 AR-related genes, shown in rank order from NEURAL-PC classification probability output. (**D**) AR.10 expression score versus NEURAL-PC binary classification. (**E**) CCP.31 expression score versus NEURAL-PC binary classification. *P* values determined from Wilcoxon’s rank sum test.

**Figure 5:**
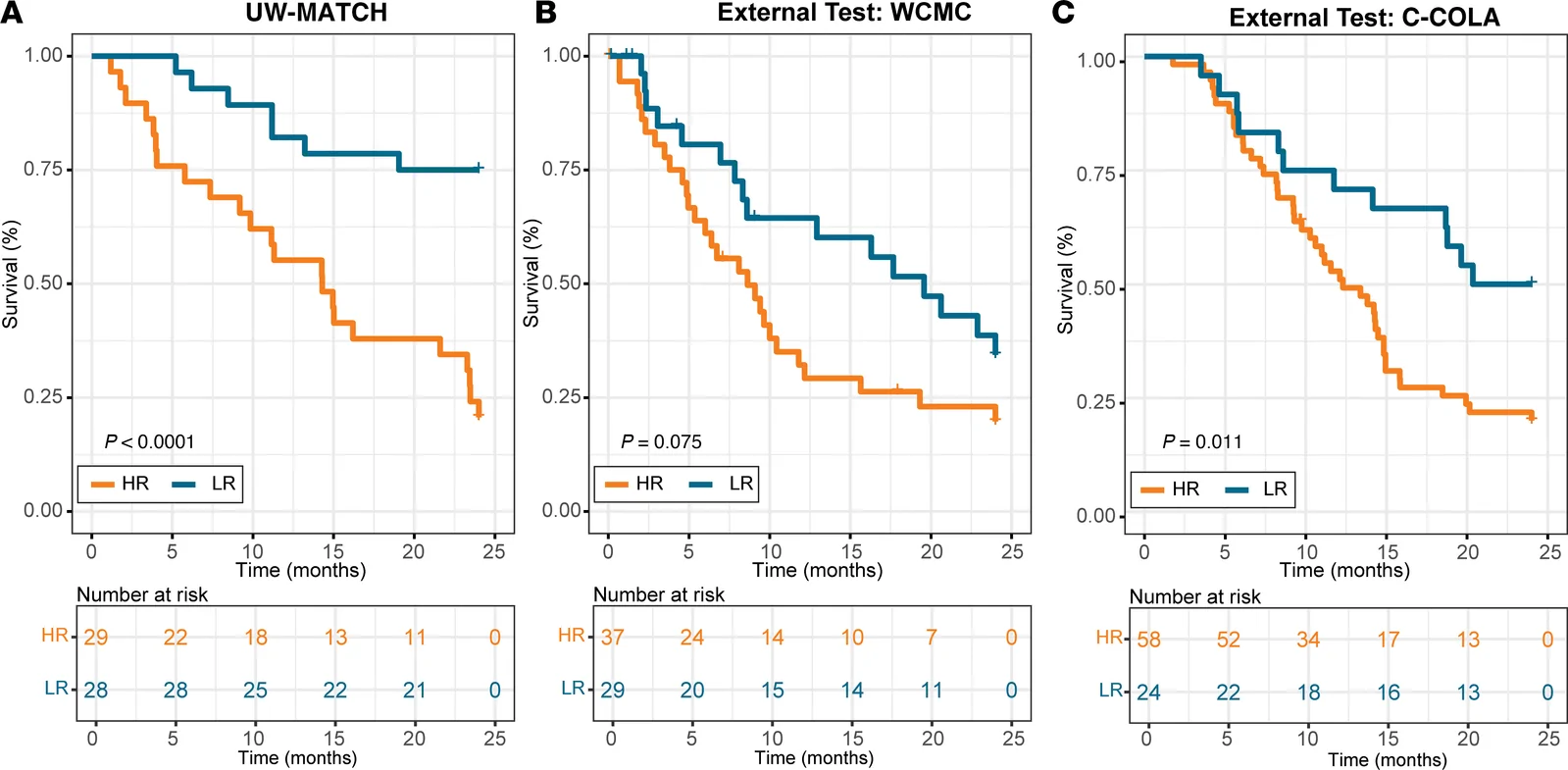
Kaplan-Meier curves for RSF model predicting likelihood of death within 2 years from biopsy. Out-of-bag predictions in training cohort (UW-MATCH) (**A**), external WCMC NEPC cohort (**B**), and external C-COLA aggressive-variant PC cohort (**C**). *P* values from log-rank test comparing groups predicted not to reach 2-year survival (high risk [HR]) and groups predicted to reach 2-year survival (low risk [LR]).

**Table 1:**
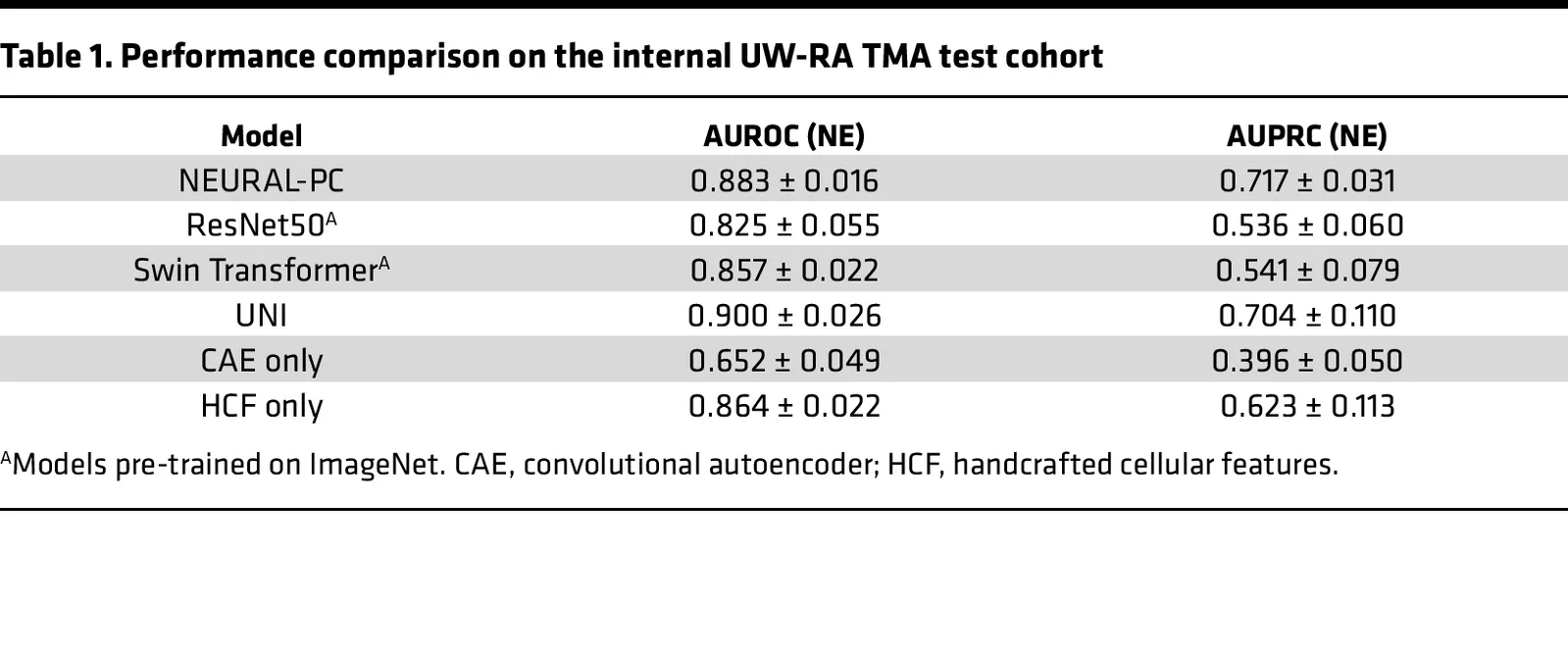
Performance comparison on the internal UW-RA TMA test cohort

**Table 2:**
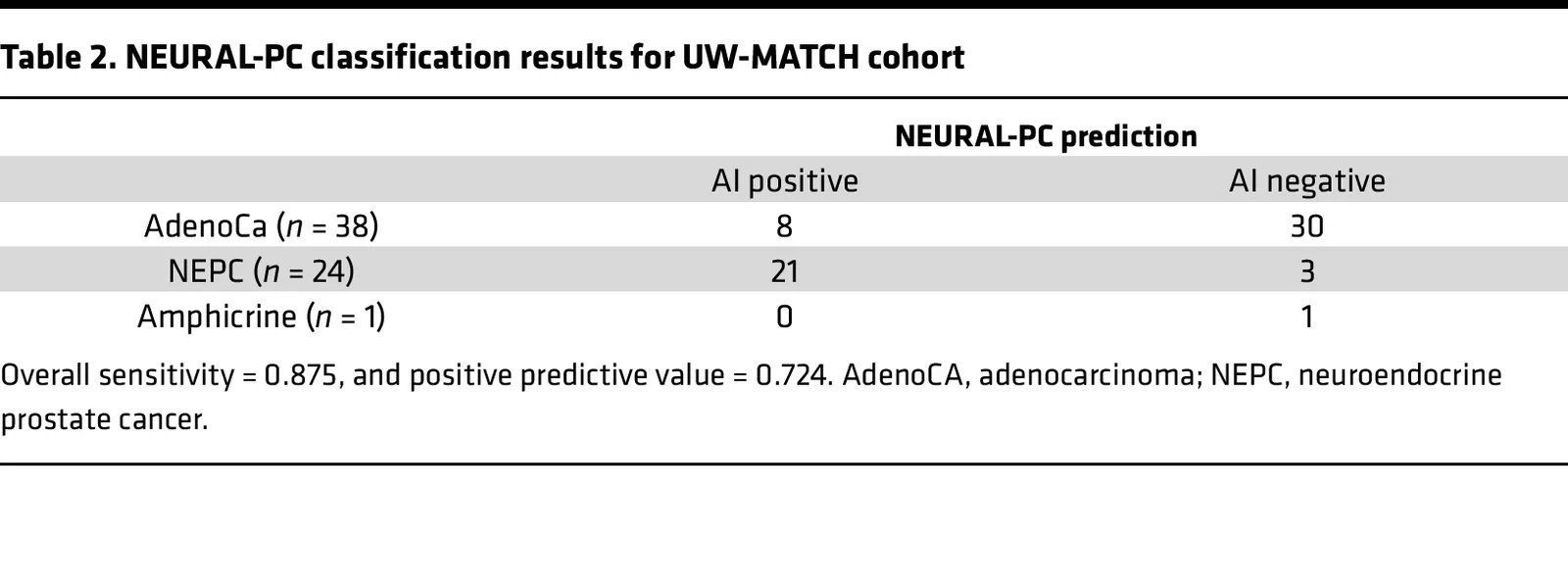
NEURAL-PC classification results for UW-MATCH cohort

**Table 3:**
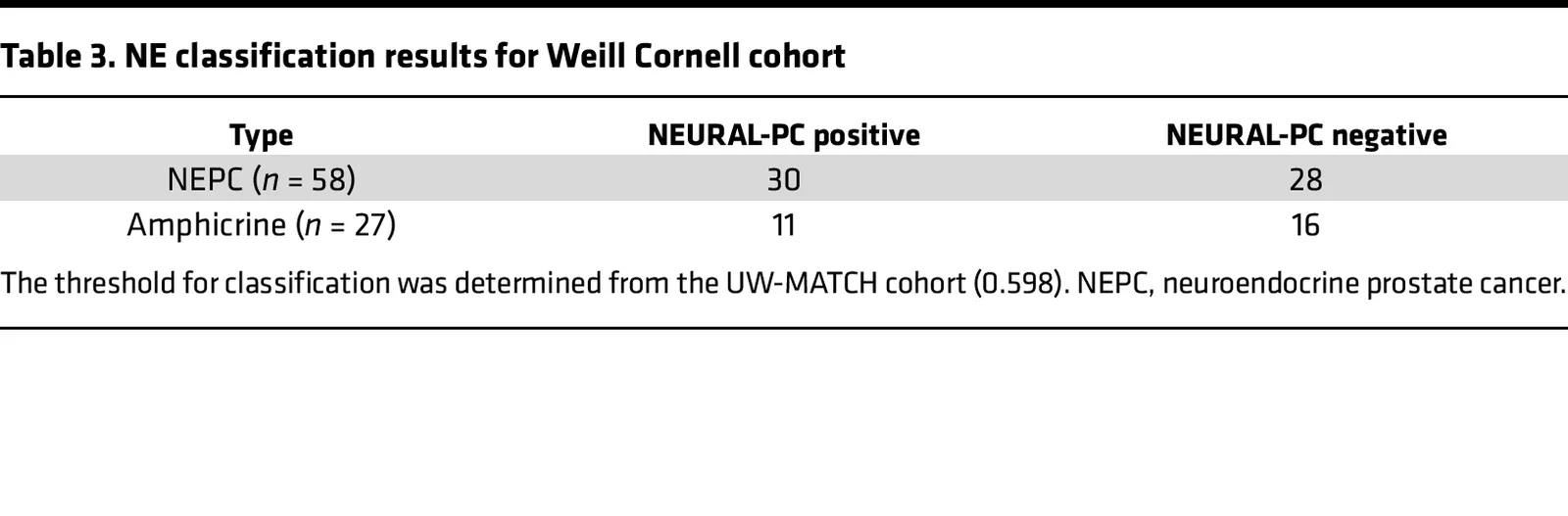
NE classification results for Weill Cornell cohort

## Version 1 08/06/2026

In-Press Preview

## Version 2 10/08/2026

Electronic publication
