# Supplementary material for: Deep learning–based histologic classifiers enable molecular subtyping of metastatic prostate cancer
Source: JCI Insight. 2026 Aug 6;11(19):e201872. doi: 10.1172/jci.insight.201872 (PMC13726411; doi:10.1172/jci.insight.201872)
Supplement: Supplementary file 1 [file jciinsight-11-201872-s204.pdf]

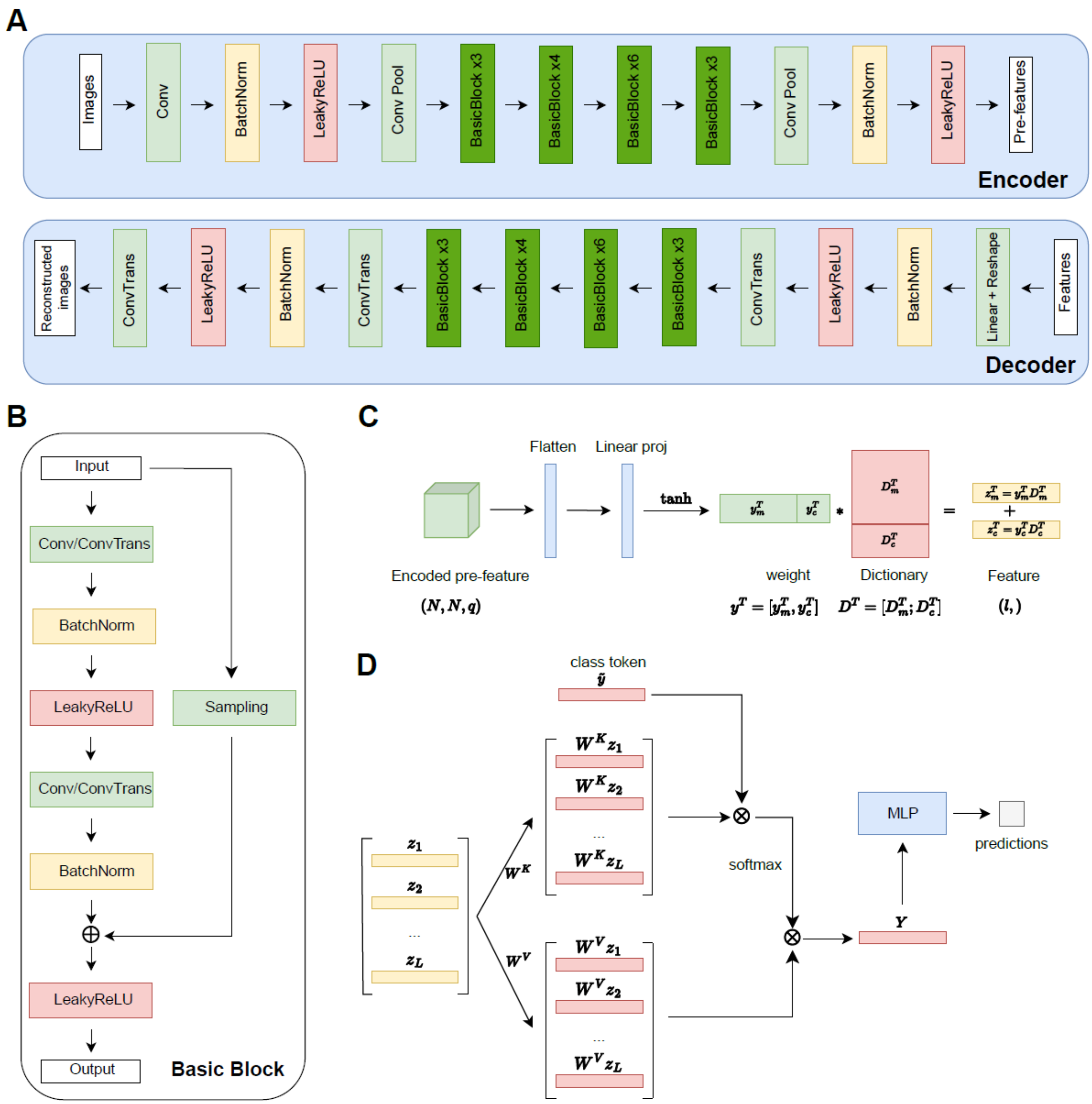

***Supplemental Figure 1. Architecture details of the proposed NEURAL-PC model. (A)** The structures of the encoder and the decoder. **(B)** The structures of the Basic Block used in the encoder and the decoder. **(C)** The structure the projector module. **(D)** The structure of the multiple instance learning module.*

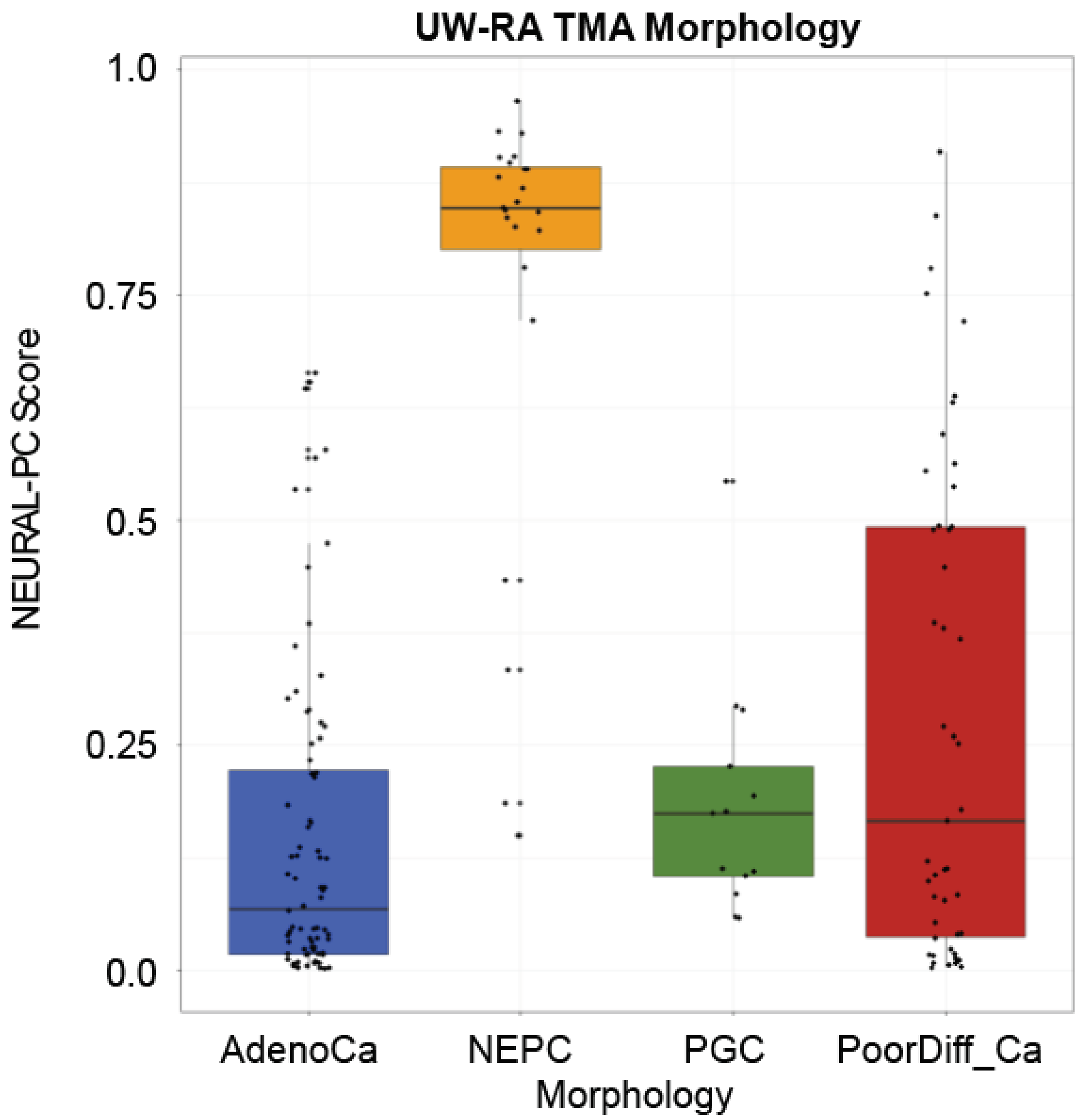

UW-RA TMA Morphology by IHC

GT	NEURAL-PC Prediction	AdenoCa	NEPC	PGC	PoorDiff_Ca
NEPC+	TP	0	19	0	6
	FN	0	2	3	3
NEPC-	FP	8	1	1	10
	TN	0	19	0	6

Supplemental Figure 2. NEURAL-PC predicted NE score distributions for UW RA TMA cores from test set with different morphological assessments by expert pathologist. *(a) continuous NEURAL-PC prediction scores. (b) Binary NEURAL-PC assessment in each morphology, with ground truth NEPC determination from IHC panel. ADC = Adenocarcinoma, NE = Neuroendocrine, PGC = carcinoma with pleomorphic giant cell features, PoorDiff_Ca = poorly differentiated carcinoma.*

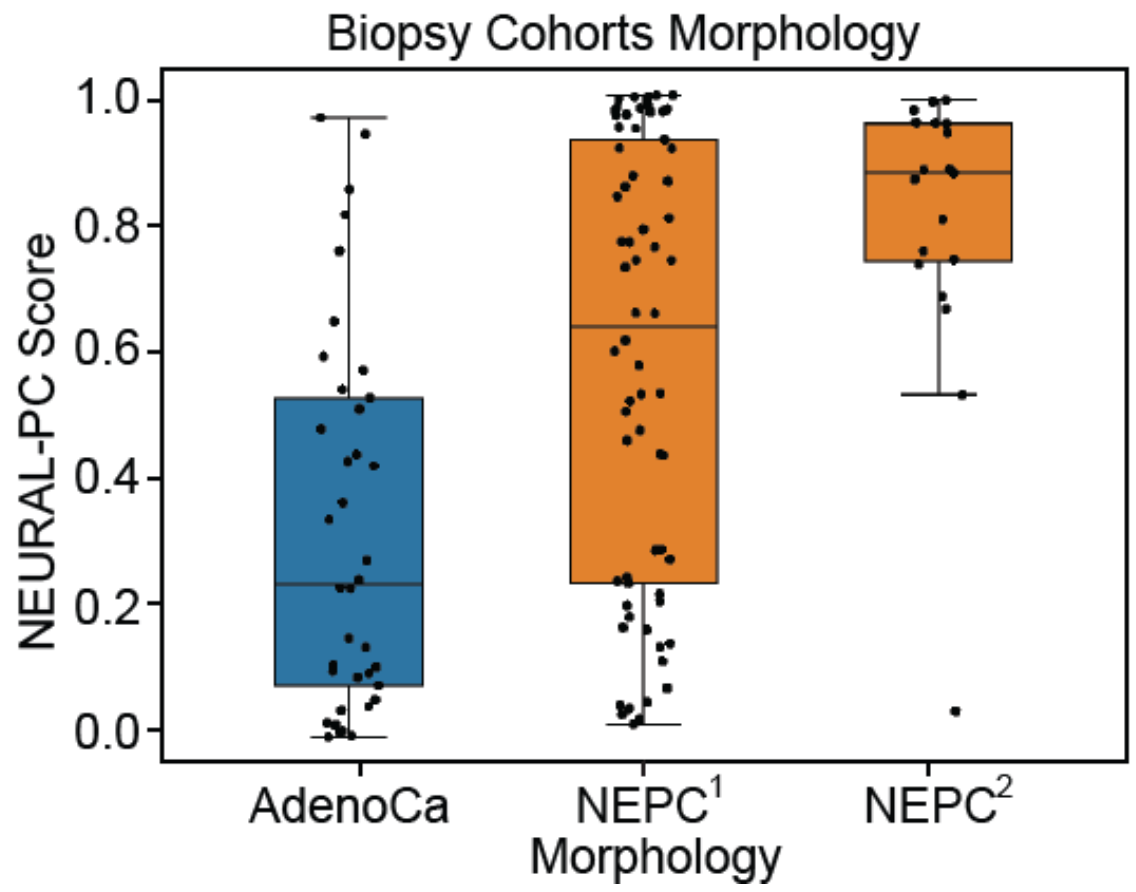

***Supplemental Figure 3*. *NEURAL-PC predicted NE score distributions for clinical biopsy cohorts*.** *AdenoCa = adenocarcinoma from UW-MATCH cohort. NEPC[1] = NEPC from WCMC cohort, samples with any NEPC-like features determined from pathology report query without strict IHC guideline adherence, NEPC[2] = NEPC from UW-MATCH cohort with strict IHC guideline adherence*[4].

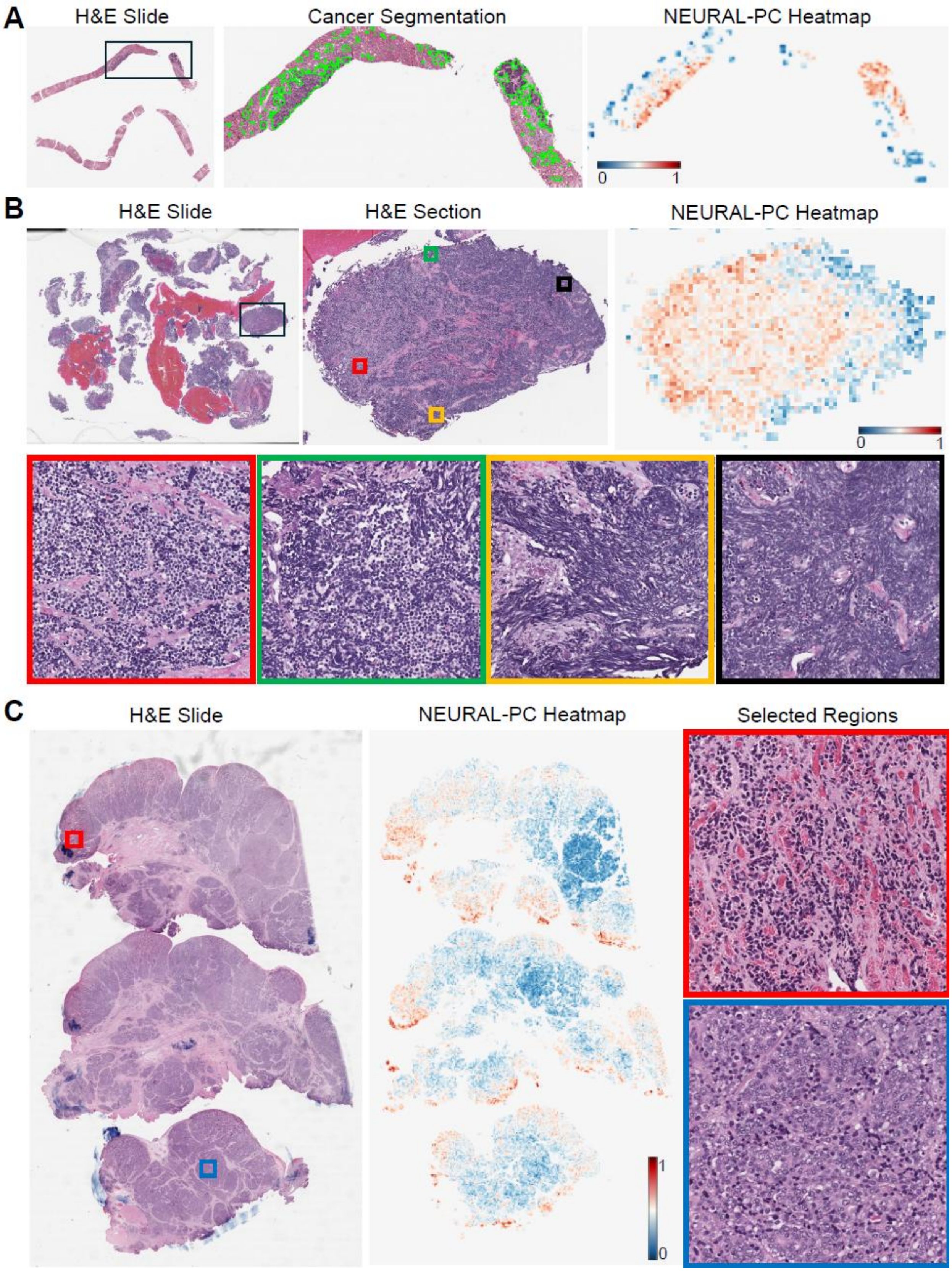

A
H&E Slide
Cancer Segmentation
NEURAL-PC Heatmap
0
1
B
H&E Slide
H&E Section
NEURAL-PC Heatmap
0
1
C
H&E Slide
NEURAL-PC Heatmap
Selected Regions
1
0

Supplemental Figure 4. Failure cases in WCMC cohort. (A) *Failure due to oversegmentation of the cancer detection algorithm. The slide was predicted as NE negative with a predicted NEURAL-PC probability of 0.155.* ***(B)*** *Failure due to image artefacts. The selected region is NE positive, but the model has low attention to the right-hand edge. Two zoomed-in views (red and green frame) from true positive NE detection regions and two zoomed-in views (orange and black frame) from false negative NE detection regions are shown. Similar artefacts in other regions of the slide cause an underestimated NE probability of 0.201 for the slide.* ***(C)*** *Failure due to small/focal NE components in large sample. While some regions show morphological characteristics consistent with NEPC (the view with red frame), the majority of the slide shows typical adenocarcinoma-like features (the view with blue frame). The whole slide is predicted as NE negative with a predicted NEURAL-PC probability of 0.231.*

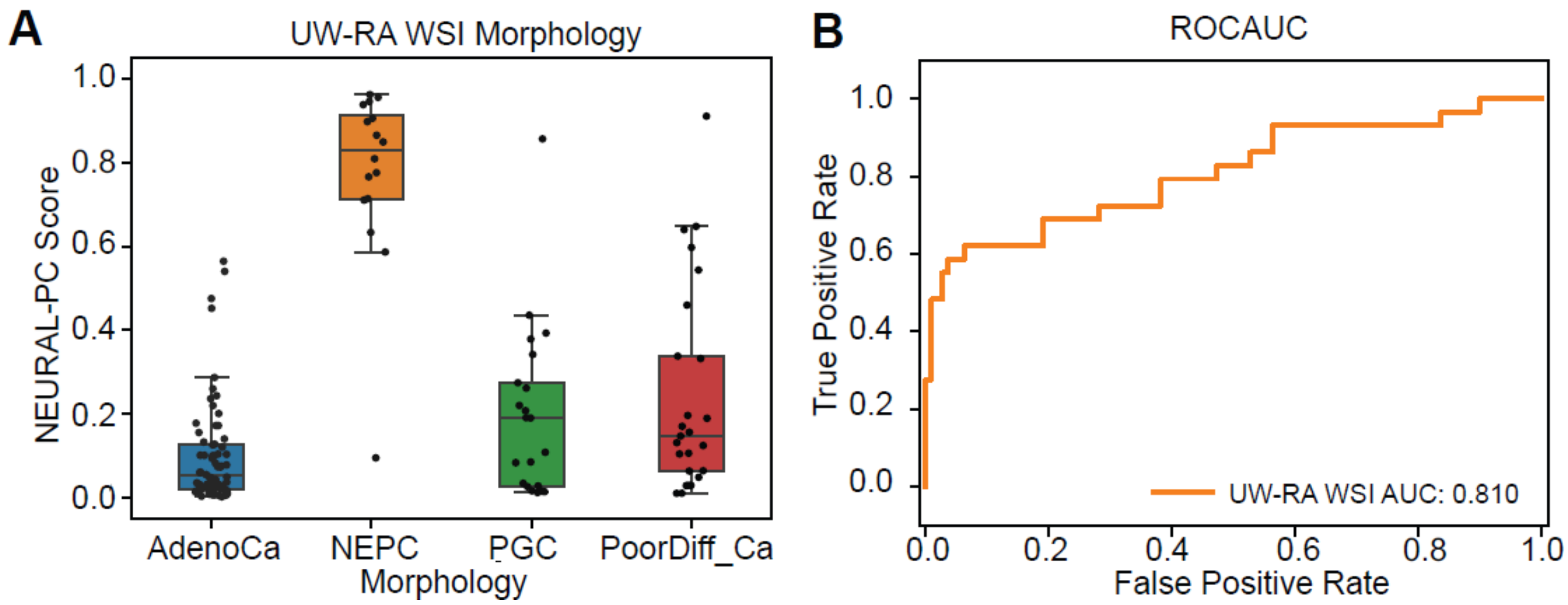

Supplemental Figure 5*. NEURAL-PC predicted NE score distributions for UW RA WSIs with different morphological assessments by expert pathologist. ADC = Adenocarcinoma, NE = Neuroendocrine, PGC = carcinoma with pleomorphic giant cell features, PoorDiff_Ca = poorly differentiated carcinoma. Scores were highest in NEPC morphologies (median: 0.829, 25%-75%: 0.713-0.914) compared to carcinoma with pleomorphic giant cell features (median: 0.189, 25%-75%: 0.027-0.274), poorly differentiated carcinoma morphologies (median: 0.146, 25%-75%: 0.062-0.337), and ADC morphologies (median: 0.051, 25%-75%: 0.018-0.126).*
